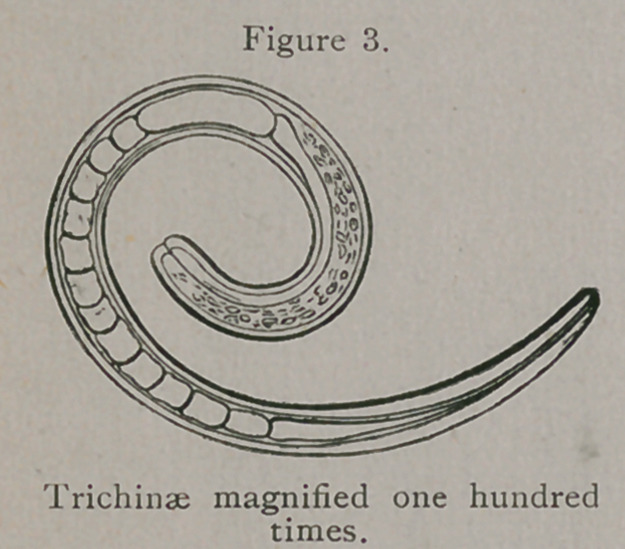# Diseased Meat and Its Consequences upon Our Health and Happiness

**Published:** 1881-01

**Authors:** Noah Cressy

**Affiliations:** Hartford, Conn.


### Art. III.—DISEASED MEAT AND ITS CONSEQUENCES UPON OUR HEALTH AND HAPPINESS.

### Trichinous Pork.

#### Part II.

There is probably no condition of diseased meat more inoffensive in general appearance, and therefore more liable to be overlooked in the culinary process, than the one caused by the presence of minute worms in the flesh of swine. Such meat is exceedingly dangerous as an article of food, and may give rise to one of the most obscure and intractable maladies that the physician has to contend with in human practice. These famous microscopic entozoa that so frequently contaminate our pork, and are known among naturalists as the *Trichina spiralis*, present in their evolution three well-marked stages of existence for us to study, which really anticipate the larva, pupa, and imago phases of development in the winged insects. The natural history of this flesh-worm, therefore, becomes not a little interesting and worthy of special notice in this connection when we endeavor to explain the precise manner of its infection, the phenomena of certain symptoms, and the ultimate cause of death in the human victim.

This parasite was first described and named by Prof. Owen* of London, in 1835, and though frequently seen by scientific observers, it was only regarded as a microscopic curiosity for more than a quarter of a century. His attention had been indirectly called to the subject some two years previously by John Hilton, demonstrator of anatomy at Guy's Hospital Medical College, who had observed a peculiar appearance of human muscle, and thought that it depended upon the formation of very small *cysticerci*. He made a communication to the Medico-chirurgical Society which was deemed worthy of publication, and this is probably the first account we have of the abode of the worm in question.*

Figure 1.

A portion of human muscle showing the cysts of the Trichinæ spiralis, natural size

Mr. Wormald, the demonstrator at the St. Bartholomew school, had frequently observed the same abnormal and speckled condition of certain muscles. The gritty sensation he had perceived, and the blunting of the edge of his scapel in dissecting, caused him one day to mention the fact to Prof. Owen. This led to some inquiries concerning the nature of these little calcified bodies in the flesh, and the distinguished anatomist at once requested a specimen for microscopic examination, as seen at Fig. 1, from the next subject he should find thus infected. It was not long before his wish was gratified, but ere he had time to investigate the matter, one of the students, now better known as Sir James Paget, dissected some of the calcareous cysts, and with the aid of a microscope, which he borrowed from Dr. Robert Brown, the well-known botanist, he actually saw the living entozoon first.†

Dr. Vogal in his description of the cyst gave it the appellation of "cocoon," believing that it was formed by the ingenuity of the parasite. Owen found each capsule to contain from one to three small hair-like worms,‡ invariably coiled up, hence he gave

* Transactions of Zoological Society, Vol. I, page 315.

† See London Medical Gazette, Vol. XI, page 605.

‡Cyclopedia Anat. and Phys., Vol. II, page 126.

it the very appropriate zoological name the parasite now bears. As this examination was made with a low magnifying power, he did not perceive that the little *helminth* had any internal organization; he therefore arranged it among the lowest of the entozoa in his new-made class *Protelmintha.* But Dr. Arthur Farre,* by his careful dissections, soon distinguished an alimentary canal, which at once elevated the parasite in the classification of naturalists to the order of nematoid worms. Yet he was unable to decide which was the anterior extremity, and for nearly fifteen years there was no advance of anatomical knowledge on the subject. It therefore remained for Prof. H. Luschka, of the Tubingen University, in 1850, to point out more accurately the internal structure. He carefully traced the digestive canal, discovered the sexual organs of the female, and conclusively proved that the mouth was situated in the pointed end of the worm, and not in the blunt extremity, as was generally believed. He described the cyst in its advanced stages, and demonstrated, for the first time, a complicated system of blood vessels, and an external membrane of connective tissue by which it is surrounded. In his observations on the vitality of the trichinæ, he found that they survived putrefaction and freezing of the muscles. Dr. Herbst, a German helminthologist, followed in this line of investigation, and his experiments on dogs actually solved the question concerning the propagation of trichinæ. He was the first to rear encapsuled flesh-worms in the muscular tissue, and claimed that in this state only they were transferable from one animal to another.

Dr. Kuchenmeister having previously shown the transformation of measles or hydatid tæniæ into tape-worms, was led to the supposition that the trichina might be a juvenile form of a known nematode; and after a series of observations, he declared that this flesh-worm was the larva of the *Trichocephalus dispar.*†

A new impluse was given to trichinal investigation in 1859, by Prof. Virchow's experiments. He fed a dog upon trichinous meat, and in four days found a large number of these nematodes

---

* London Gazette, Dec., 1835.

† Animal and Vegetable Parasites, Sydenham Ed., Vol. I, page 321.

fully developed and sexually mature in the intestines, but he failed to observe the migration of the new-born worms which Herbst had previously demonstrated. This was owing partially to his having killed the dog too early, and also from the fact that he selected an old animal for the experiment, through whose firm tissues the young trichinæ scarcely ever penetrate.

Dr. Zenker of the Dresden Medical School supplemented these observations, and threw much light upon the subject in a medical point of view. He found, upon microscopic examinations, free and living trichinæ in the muscles of a servant girl who died in the hospital, at the age of twenty, of what was supposed to be a typhoid fever. She was taken ill January 12, 1860, and fell a victim to this strange malady within a month. Her symptoms were severe, and in some respect resembled rheumatism, with painful swellings of the limbs. The history of the case, therefore, was of more than usual interest to the profession, and excited not a little clinical inquiry, but no one mistrusted the cause of the trouble. It was soon ascertained, however, after Zenker's post-mortem disclosure, that she had assisted in the making of sausage on the 21st of December previously, and that she had partaken of some of the raw meat only a few days before her illness. This led to his well-known investigation on the nature and pathology of trichinosis, which has been so extensively published to the world, and will ever crown his life with honor. The discovery of this violent parasitic disease in man aroused at once the zeal of professional experts and veterinarians, and was the dawn of a new era in sanitary science.

Prof. R. Leuchart,* of Giessen, followed up the researches on the embryology of the parasite ; he made a series of experiments on trichinal infection that were very comprehensive, and did much to advance the science of helminthology. He corrected his own previously expressed opinion on the validity of Kuchenmeister's observation on the transformation of the flesh-worm into trichocephalus, and thus confirmed Virchow. He also showed that the young trichinæ in the intestines became the

* For a summary of his views see Burk's translation in Quar. Jour. of Microscopical Science, Vol. VIII., page 168

encysted worm in the muscles, and he believed that they reached there by migration through the tissues, while others claim that the distribution of the trichinæ over the body in so short a time can only be effected through the circulation of the blood.

Dr. Thudichum,* who has made extensive researches on the parasitic diseases of food animals, says concerning this worm, that the red voluntary muscles are the "promised land" of the trichinæ. There they migrate, grow, and enshrine themselves. Although the young trichinæ, on the seventh day and later after infection, are found in almost all the organs of the body, yet they do not grow or become encapsuled in any other tissue. The trichinæ arrive in the muscular tissue with the blood. The diameter of the smallest capillaries in the muscles is much less than the diameter of the young trichinæ, so they are certain to be arrested. They then penetrate the simple or double coats of the muscles, and are at once in the interstitial spaces between the muscular fibres. Many trichinæ unquestionably never enter the sarcolemma, and become encysted, but when they do the fibres become permanently destroyed. At the end of the third week after immigration, the inflammatory irritation of the muscular fibre has reached its highest point, the trichina is nearly full grown, and becomes fixed to the spot where it is to be encapsuled. Several of these worms may wander in the same track, and ultimately be enclosed in one lump of exuded matter.

It is in the encysted state, as seen at Figure 2, that the trichina is transported from one flesh-eating animal to another. Pigs are not born with these entozoa, but get them in some kind of food, probably from the flesh of rats and mice, and when once swallowed by the hog or other animal, the gastric juice, in the process of digestion, soon dissolves this albuminono-cretaceous cyst, when the parasite will be liberated from its prison life, and in a few days becomes a full-grown worm.

---

* See his able paper on the subject in the 7th Public Health Report of the Privy Council, London, 1865.

Figure 2.

A portion of ham, showing two cysts of the Trichinæ spiralis enclosed, slightly magnified.

The sexually mature female, according to Prof. Cobbold,* is one-eighth of an inch in length, while the male is only about two-thirds that size. The female is ovo-viviparous, and thus brings forth its young alive within the stomach and intestines. The young trichinæ begin at once to migrate from the bowels and perambulate the entire system of voluntary muscles; at last they become encased, and there remain for ever at rest, until they, perchance, shall have been eaten by some other animal, when they, in turn, will be set free, and thus complete their final destiny.

This parasite, which undoubtedly infects a large number of animals, has frequently been found in the rat, mouse, cat, hedgehog, fox, mole, and hog, and is liable to be transmitted from one carnivorous animal to another through the meat. The Commission of the Royal College of Physicians of Vienna report that the main source of the infection in the hog is from the rat, and nearly one-half of all these vermin examined in Moravia were found infected with the encysted trichinæ; and it is not improbable, as Fleming observes, that the rats were primarily infected, and have thus transmitted these parasites from one generation to another by virtue of their carnivorous habit, at times, to devour each other.†

Figure 3.

Trichinæ magnified one hundred times.

Although the swine of every land may occasionally be infected with this noxious parasite, still the frequency of its transmission will depend in a great measure upon the habits of the people. In those countries where the practice of eating raw pork and sausages so extensively prevails, of course the parasites contained in the flesh will be transported to the human stomach

* See his Entozoa—An introduction to the study of Helmintholy, with Supplement. London, 1869.

† Veterinary Sanitary Science.

unmolested, but no fears need be anticipated from even the free use of pork if it has been subjected to a sufficient degree of heat in the process of cooking, to destroy every germ of animal life; then it would be as harmless from this cause as fish, beef, or venison.

The ravages of this loathsome malady from the use of diseased pork are not confined to any country, and I believe it prevails more extensively than is generally supposed. Dr. George Sutton, of Aurora, Indiana, who has been examining pork killed in the State, says he has found from three to sixteen per cent. of the hogs affected with this disease—differing in various localities—and that, taking the rate at four per cent., we have put upon the market, from the Western States, 221,484 diseased hogs, or about 44,296,800 pounds of infected meat, every ounce of which might produce disease.* And the Committee of the Chicago Academy of Science has shown that the percentage of swine infected by the trichina in the Western States is greater than in Germany. Still, the disease is of rare occurrence on this side of the Atlantic, compared to the old country; and we can ascribe no cause for the greater prevalence of this disease in Germany, except it be the habit of eating their ham or sausage in the raw, or uncooked state.

The symptoms of trichinous infections in man will depend largely upon the quantity of diseased meat that has been eaten, and also upon the stage of the malady. At first it is marked by local irritation within the intestinal track, before the worms begin to migrate. This gives rise to nausea, loss of appetite, inflammation of the mucous surface of the bowels, and diarrhœa. Peritonitis may sometimes occur from the perforation of the intestinal walls.

The second stage is characterized by general symptoms, muscular pains, rheumatism, etc., occasioned by the migration of the worms in the various parts of the body. There is great soreness, œdema, and stiffness of the muscles. Lassitude and profuse sweating not unusually occur in severe cases, and in this

* A report on Trichinosis, from the Transactions of the Ind. State Medical Society, 1875.

respect it resembles typhoid fever, for which it has many times been mistaken. This stage commences in about ten days from the first illness, and lasts four or five weeks.

In the third phase of the malady the trichinæ have become encysted, the fever, soreness, and inflammation begin to abate, and the patient is in a fair way to recover. In many cases there is a complete restoration to health again, but often it leaves the system in a very prostrate condition, according to the amount of muscular lesion that has taken place.

Thus our only safety from the use of pork, which is always more or less liable to contain trichinæ in any portion of the country, is thorough *cooking*. Salting and smoking, unless long continued, has but little effect upon the vitality of these parasites. Raw ham or sausage should never be allowed upon a sanitary bill of fare; and even boiled ham, when large and fashionably prepared, as seen in many of our eating saloons to-day, not unfrequently contains these living worms. Hence our lives may be prolonged and our health improved by more attention being given to the domestic duties of the household. Then will all meats be served upon our table in a manner both to nourish and promote our happiness.

### MEASLY PORK AND BEEF.

This condition of diseased meat is caused by the presence of a larval form of a tape-worm known as a *scolex* or a *hydatid* in the flesh, thus giving it the spotted appearance of the measles, and consequently this term has been applied to the parasite. There are several points of resemblance between the life-history of the tape-worm and the trichina. The measles are the young entozoa in the encysted state and likewise are received into the stomach with the meat, when they become liberated in the process of digestion and grow into the mature parasite. But tape-worms do not multiply like trichina in the human body. Each one comes from a hydatid measle that has been eaten.

The posterior segments of the tape-worm ripen and fall off, and are known as progottides. Each one is sexually complete, a hermaphrodite, and contains a multitude of mature ova; and when eaten by other animals the eggs, set free in the stomach, readily hatch, and the embryo worms with their six hooklets or spines arranged about the head soon perforate the intestinal walls, enter the blood-vessels, and are transported by the circulation to all parts of the system. Being therefore a foreign body in the flesh, inflammation ensues, and they are soon enclosed by an exudation thrown out for protection, and thus a cyst is formed where they develop into a measle, or the *cysticurcus celulosæ* of early authors.

The larval cestode which infests the measly pork is the sexually undeveloped progeny of the armed tape-worm known as the *Tænia solium* of Linnæus. And while the pork measles have been found in the flesh of other animals, the adult parasite seems to claim no other host than the human intestine. The pork tape-worm is probably the best known and by far the most prevalent. In fact, this intestinal parasite is only too frequently the pork-eater's guest. It is therefore obvious that the measly condition of the flesh of swine is the real source of danger.

Hence the necessity for more precaution on our part in rearing these animals for home use, or for the general market. Thus, a person harboring a single tape-worm may be the means of contaminating many hogs, for each mature segment cast off contains thousands of eggs ready to hatch when taken into the stomach of other animals. Consequently, swine should never be allowed access to privy dwellings, nor to compost heaps containing night-soil. Better sanitary regulations are therefore demanded even in the homes of the affluent.

The beef measle, when eaten by man, develops into the *Taenia mediocanellata,* which Kuchenmeister first described and named.* This tape-worm was looked upon for a long time simply as an unarmed variety, but it is now known to be a distinct species. It is larger than the other, and the joints are wider. This parasite occurs among beef-eating people, and is quite common in America and European countries. In fact, it prevails most extensively among the Jews and Mahommedan nations, where pork is not

---

* Animal Parasites, vol. 1, page 133.

used, thus showing conclusively that the broad, unarmed tape-worm comes from the use of measly beef and veal.*

The same sanitary measures will be required to protect the cattle against this larval infection. Their voracious habits sometimes will cause them to eat strange substances. Cattle should therefore not be permitted to frequent those places where human ordure has been left, or to drink from dead ponds that receive the wash from dwellings, especially when it is known that people in the neighborhood are infested with the beef tape-worm.

Though our staple articles of meat may be affected with these larval parasites, yet thorough cooking renders all such flesh perfectly harmless. Raw pork, veal, and beef are dangerous articles of food unless a microscopic examination has been made to determine the possibility of any parasitic infection. And Prof. T. Spencer Cobbold of the Royal Veterinary College, who by his original researches has contributed so largely to our professional literature on this subject, has announced the discovery of a mutton measle, differing in some respects from those other two; but whether the resulting tape-worm will make its abode in man, and thus render the favorite flesh of sheep a diseased article also for human use, has not been determined.

The mature ova of the *Tænia solium*, when taken into the human body with food or drink, develop into the measle quite as readily as in the hog, and thus our own flesh is liable to become the bearer of these larval parasites, a single one of which may prove fatal. Hence, we should strive to guard against all such possible contingencies.†

### BRAXY MUTTON AND BLACKLEG VEAL.

In this type of diseased meat we have a virulent blood poison to deal with which infects the entire carcass; and accordingly all animals dying from an anthrax fever in any form should be condemned. Yet we find that the flesh of braxied sheep is a favorite

---

* For further account of these measles see Prof Verrill's able paper on Internal Parasites, in the Secretary's Report, 1869.

† See supplement to his large work on the Entozoa, 1869; and also his new Manual on the Parasites of Man and Animals. London, 1879.

article with many, and some even prefer it to the best dressed mutton in the market, claiming that it is more wholesome and easier of digestion. Such a taste of course must be an acquired one, like that of the epicures who seem to relish their game the best when in a state of decomposition. And some forms of braxy mutton are in a similar condition, being soft and putrid many times as soon as the animal is dead.* Such practice is much to be reprobated says a practical shepherd,† although it is almost universal among those connected with the stock in every district where the disease prevails, and in some places large quantities of hams are salted and dried; and from the amount consumed in certain localities there is no doubt but that it is the cause of many blood poisons among the people.

Instances are not wanting where blackleg veal and other anthrax varieties of flesh have been eaten with impunity. There is evidently a great difference in the intensity of the malady. Sometimes the poison may not be fully elaborated, and consequently the virulency of the meat will be less energetic. Thorough cooking may do much to destroy these infectious properties, yet the extensive alterations that have taken place in the tissues and the rapidity with which putrefaction sets in are sufficient reasons, on sanitary grounds, to absolutely prohibit the use of all such diseased meat.

The nature, history, and symptoms of two very malignant anthrax maladies in cattle—splenic apoplexy and charbon—will be found treated at some length in relation to the diseased meat question in my third report, as State Veterinary Surgeon, on the Diseases of Domestic Animals within your State.‡

### THE LUNG PLAGUE.

The question of the wholesomeness of meat from animals affected with pleuro-pneumonia is one of much moment to us

---

* See Robertson's Prize Essay on Braxy in Transactions of the Highland and Agricultural Society of Scotland, Vol. 19.

† Cowan's Essay on Braxy, in the 18th Vol. of the above transactions.

‡ In the Secretary's Agricultural Report of 1873. See also Fleming's Veterinary Sanitary Science.

to-day, and in view of the increasing prevalence of the malady in this country, it is worthy of our consideration and of our expressed opinions for the public good.

Since the first introduction of the lung plague in 1843, the herds of the New England and Middle States have not been free from its local ravages. During its history of thirty-seven years upon our virgin soil, the germs of this contagious malady have become so effectually disseminated along the Atlantic coast, that fresh and unexpected outbreaks are now of frequent occurrence, especially in the vicinity of New York, where the disease first made its appearance.

The western movement of our thorough-bred stock of late years has opened new channels for the development of the disease; and unless immediate action is taken by Congress to intercept the impending scourge, these potent germs will ere long be found lurking among the vast herds upon the plains, if not already wafted there by the trend of civilization.

The cattle of Connecticut, for some time past, have been more or less affected by this disease, and thrice have we been called upon as a Commission in the last ten years to stay its progress. And still there is more work to be done which the next Legislature, in its wisdom and economy, cannot ignore. And should pleuro-pneumonia, the dreaded plague of our land, ever become through political neglect a general epizootic, and thus sweep across the continent, decimating our herds upon the hilltops and plains, we are not without a bright sanitary consolation in such an event to cheer the poor and broken-hearted, for the meat from thousands upon thousands of our fine animals, that must of necessity fall victims in its march, if slaughtered at an early stage of the malady, could be utilized in a great measure to feed the hungry and needy without a fear of dangerous consequences to the health of our panic-stricken millions.

Thus our great dread of the extension of the lung plague is not on account of any infectious condition of the meat, for millions of those affected animals, according to Fleming, have been consumed as human food in various parts of the world, and no vil results have been known to follow. In Great Britain and

France, there has been for years a regular trade with the butchers in cattle affected with the contagious form of this disease, and yet the sanitary condition of the people remains unimpaired. But it is the immense loss to our live stock property that would be entailed by such a calamity.

### CONCLUSION.

Of the other maladies which are liable to effect our meat supply, the foot and mouth disease is one that is much to be dreaded. And though the flesh of such affected animals is believed to be harmless, yet the milk, under certain conditions, is a dangerous article of food. But observers are not agreed in relation to the matter. The same is true in regard to hog cholera and several other forms of disease, the pathology of which is not sufficiently well understood to enable us, at the present time, to solve the various sanitary questions here involved.

Hence the necessity for a competent veterinary inspector in every State, whose professional duties to the public have already been outlined, in the consideration of our theme. And as we have seen that our health and happiness is frequently involved in the very meat we eat, such an officer, in watching the development and progress of these infectious maladies among our food-producing animals would thus serve as the true guardian of human welfare.

NOAH CRESSY, M. D., Ph. D.
*Hartford, Conn.*

## Figures and Tables

**Figure 1. f1:**
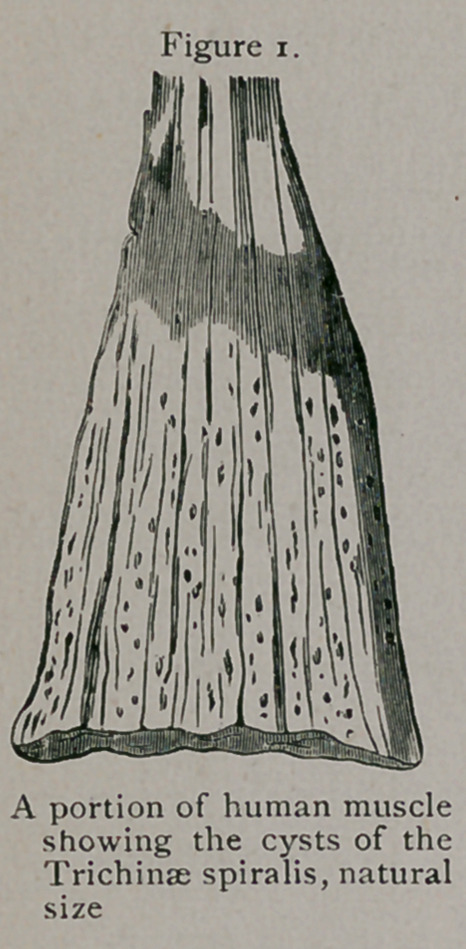


**Figure 2. f2:**
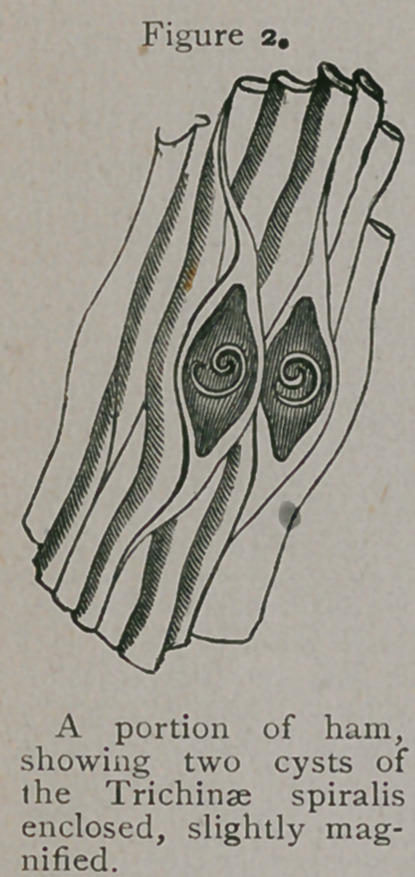


**Figure 3. f3:**